# Factors Affecting Mammalian Occupancy and Species Richness in Annapurna Conservation Area, Nepal

**DOI:** 10.1002/ece3.70572

**Published:** 2024-11-18

**Authors:** Yadav Ghimirey, Raju Acharya, Jeffrey Mintz

**Affiliations:** ^1^ Department of Wildlife Ecology and Conservation University of Florida Gainesville Florida USA; ^2^ Friends of Nature Kathmandu Nepal; ^3^ School of Natural Resources and Environment University of Florida Gainesville Florida USA

**Keywords:** Bayesian hierarchical model, detection probability, imperfect detection, mammal community, multi‐species occupancy models, species richness

## Abstract

Species richness is an important metric used for undertaking conservation management decisions. However, species richness estimates are influenced by species detection probabilities, with potential to entirely overlook species during surveys. Occupancy models which account for imperfect detection provide unbiased estimates, ensuring accurate estimates of richness. We carried out a camera trap survey in the mountains of north‐central Nepal during 2017 and documented a total of 21 mammal species. Here, we used multi‐species occupancy models within a Bayesian hierarchical framework to reassess our initial species richness estimate and to understand the influence of environmental covariates on occupancy and species richness of mammals in the area. Our model estimated the mean species richness was ~26 species (95% CRI: 21–36 species), suggesting we might have missed ~5 species during the survey. The mean probability of occupancy and detection of mammal species were estimated to be $0.28\;\left(95\%\mathrm{CRI}:0.08–0.46\right)$ and 0.02 (95% CRI:0.01–0.03) respectively. Mammalian species richness of the area had an anticipated positive relationship with tree canopy cover $\left(\beta =1.908,95\%\mathrm{CI}=0.989–2.827,p=1.95e-04\right)$ though its positive relationship with anthropogenic disturbance was surprising $\left(\beta =1.339,95\%\mathrm{CI}=0.334–2.344,p=0.012\right)$. Mammalian species richness had a quadratic relationship with elevation as is expected. This research contributes to baseline information of the mammal community ecology in north‐central Nepal and supports the need for future multi‐season surveys to understand the influence of temporal factors on mammalian community and species richness in the area.

## Graphical abstract

We used multi‐species occupancy models to camera trap data from central Nepal to estimate mammalian species richness and to understand the influence of environmental covariates on their occupancy. We estimated the mean species richness of ~26 species. The mean probability of occupancy and detection were estimated to be 0.28 and 0.02, respectively. Mammalian species richness of the area had an expected positive relationship with tree canopy cover though its positive relationship with anthropogenic disturbance was surprising.

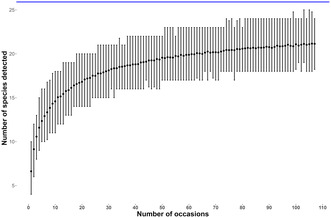

## 1 Introduction

Species richness, which refers to the total number of species in a given area at any given point in time, holds significant importance in ecology as it is considered a premier indicator of biodiversity (Chaudhary et al. 2022; Peet 1974). Given that it is an important conservation indicator, many ecological and conservation studies aim to reliably estimate species richness (Gotelli and Colwell 2010; Weber, Hintermann, and Zangger 2004) using methods such as species accumulation distributions, species accumulation curves, or nonparametric estimators (Walther and Morand 1998). However, completely documenting all species in an area during surveys is practically impossible due to imperfect species detection as many species will likely remain undetected in surveys (Iknayan et al. 2014; Nichols et al. 1998). Even for plants this remains true, as survey and site conditions contribute to this challenge (Chen et al. 2013). As a result, estimates of species richness that don't consider imperfect detection are flawed and unreliable (Guillera‐Arroita, Kery, and Lahoz‐Monfort 2019). Nonetheless, many studies assume perfect species detection when estimating species richness (Espinar 2006; Hiiesalu et al. 2014). To address this issue of imperfect detection, occupancy modeling is being increasingly used, which incorporates either temporal or spatial replicates to track species detection history (MacKenzie et al. 2017, 2002). Occupancy models have been designed to estimate species richness in an area based on the detection histories of all observed species and a few augmented species (Dorazio et al. 2006). This approach helps overcome the challenges posed by imperfect detection and provides more accurate insights into the true species richness of an area. Despite the availability of strong statistical frameworks to address the issue of imperfect detection, many recent studies conducted in Nepal still estimate species richness, assuming that the detections are perfect (Baniya et al. 2010; Gautam et al. 2016; Khatiwada and Haugaasen 2015), with only a few exceptions (Regmi et al. 2023; Thapa et al. 2022).

We conducted a camera trap survey during the winter of 2017 season in the contiguous subtropical and temperate forests of the Annapurna Conservation Area (hereafter ACA), located in north‐central Nepal. The primary objective was to assess the status of felids and their prey species in the region that can be used as a baseline for future studies. To analyze the data, we used multi‐species occupancy models within a Bayesian hierarchical modeling framework to estimate species‐specific occupancy and detection probabilities as well as mammal community occupancy (Kéry and Royle 2015; MacKenzie et al. 2017; Nichols et al. 1998). Additionally, we employed the single‐season, multi‐species occupancy model framework to estimate the actual species richness of the area (Dorazio et al. 2006; Kéry and Royle 2015). We also assessed the influence of environmental and anthropogenic factors on species‐specific occupancy probability and species richness considering these factors are known to influence these parameters (MacKenzie et al. 2017; Teixeira‐Santos et al. 2020). While we know that these parameters are influenced by environmental and anthropogenic factors, the exact direction of this influence tends to differ based on the spatial scale, geographic location, and taxonomic groups involved (MacKenzie et al. 2017). Currently, very little effort have gone to understand the mammalian community as a whole in the subtropical and temperate forests of the Nepalese mountains (but see Regmi et al. 2023; Thapa et al. 2022). Such analysis would provide an important picture of the status of mammalian community in ACA which represents Nepalese mountains. Hence, our rationale for this analysis was to estimate the number of possibly missed species during survey and also assess the influence of covariates on species richness and species‐specific detection and occupancy probability. This would also help to facilitate future research and conservation initiatives in the area as well as encourage replication in other regions of the country. As a result, we also aimed to discern the relationship between the species occupancy and detection probabilities as well as the mammalian species richness with the environmental variables of the area.

Potential concern arises when interpreting the status of the overall mammal community based on camera trapping efforts that focus on either a species or species guild, as this may introduce bias due to variations in occupancy and detection probabilities (Devarajan, Morelli, and Tenan 2020). In this case, our survey represents habitat favored by felids within the ACA, particularly those portions accessible to humans via trail networks. Nonetheless, it is important to acknowledge the challenges of conducting targeted surveys for all species in a community, given logistical constraints and funding limitations (Rahman, Sitorus, and Condro 2022). Therefore, analyzing data resulting from single species or guild surveys using statistically robust methods can be valuable in understanding the status of multiple species within a community (Chaudhary et al. 2022; Rasphone et al. 2019), offering critical information for future community‐level monitoring.

## 2 Materials and Methods

### 2.1 Study Area

**FIGURE 1:**
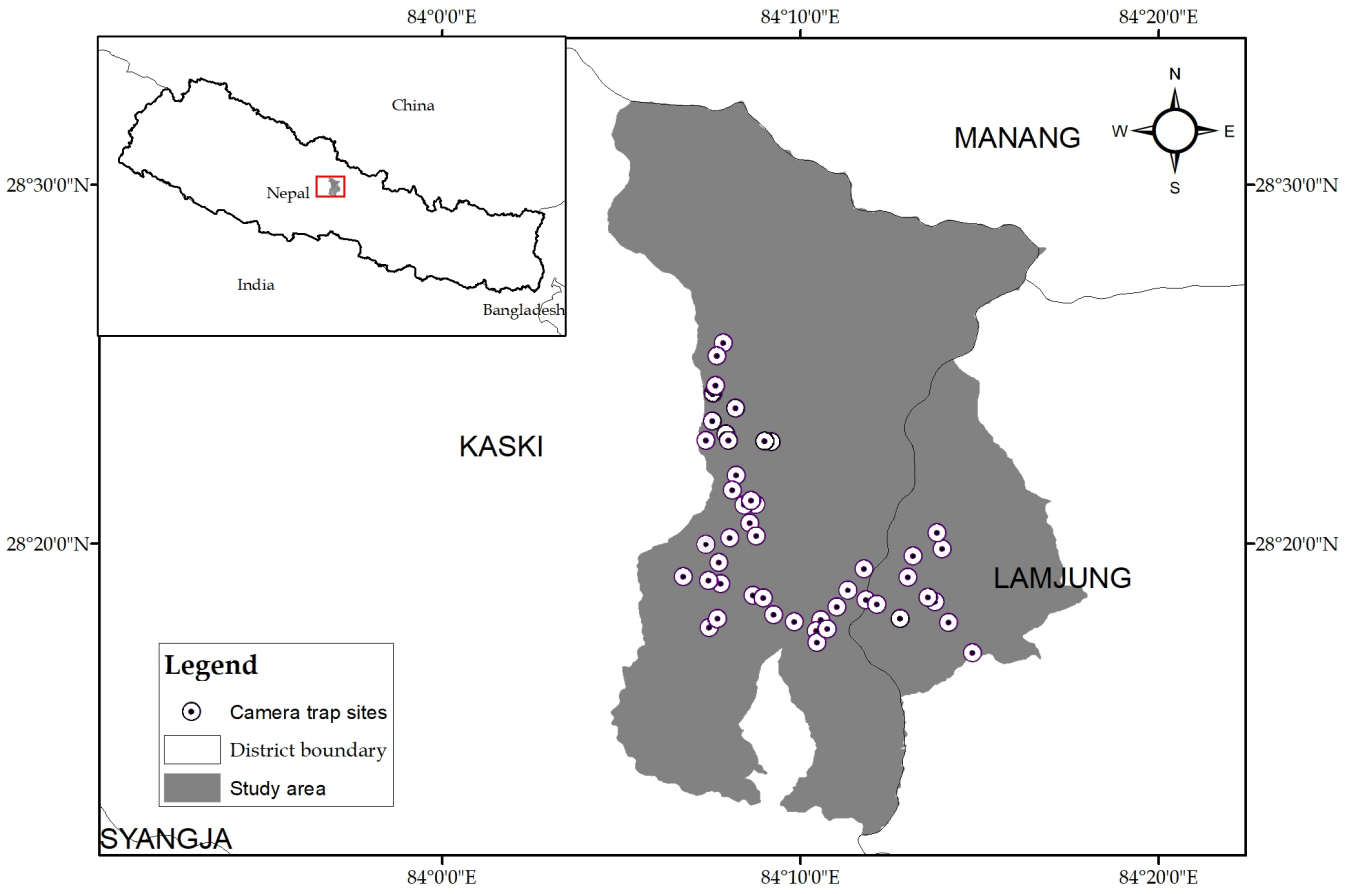
The study area was located in the contiguous forests between Kaski and Lamjung districts. Circles with black dots in the centre indicate camera trap locations.

The study was conducted in the contiguous forests between Kaski and Lamjung districts within the lower part of the ACA (Figure 1) in the central Himalayas, one of the world's biodiversity hotspots (Mittermeier et al. 2004; NTNC 2009). This region comprises both subtropical and temperate forests, commonly represented by needlewood *Schima wallichi—*chestnut *Castanopsis indica* and oak *Quercus* spp. – *Rhododendron* spp. associations respectively (NTNC 2009). The area is home to several important wildlife species, including the common leopard *Panthera pardus* , clouded leopard *Neofelis nebulosa* , Himalayan black bear *Ursus thibetanus* , Asiatic wild dog *Cuon alpinus* , Himalayan serow *Capricornis thar* , and northern barking deer *Muntiacus vaginalis* (Ghimirey et al. 2018).

Around 800 households inhabit the study region, the majority of whom rely on farming and livestock herding for their livelihood. The local communities heavily depend on the forest for resources like timber, fuelwood, medicinal plants, and other non‐timber forest products (NTFP). Additionally, illegal hunting of wildlife is a serious issue in the area (NTNC 2009). Unfortunately, this has led to forest degradation, conflicts between humans and wildlife, and retaliatory killings, which pose substantial yet unquantified threats to the wildlife in the region.

### 2.2 Methods

#### 2.2.1 Data Collection

The area below the elevation of 3000 m in the study area was delineated using QGIS 2.12 (QGIS Development Team, 2024) and overlaid with regularly spaced points, each 1 km apart. This ensured that the placement of camera stations allowed for spatial coverage and accounted for the assumption of independent detections. A total of 53 camera stations were selected, excluding points that were inaccessible, and favoring locations known to be used by felids. The total area covered by the camera traps amounted to approximately 130 sq. km. Camera traps were active between 31 December 2016 and 17 April 2017, with temporal coverage ranging from a minimum of 74 days to a maximum of 107 days, depending on when the cameras were initially placed and subsequently retrieved.

Regional wildlife and human trails were traversed to look for evidence of mammal species (pugmarks, scats, claw marks, and scrapes) before setting up the camera traps. While the target species were wild felids and their prey, we were unable to identify the species' signs in several situations. This prompted us to look for spots in prime locations that were more likely to be visited by most mammal species. For example, sites at trail crossings, ridgelines, trail‐ridgeline crossovers, wildlife corridors, hunting trails, and water sources were selected. Hunters and cattle herders were also consulted to supplement the information on mammal occurrence patterns and the areas with the highest likelihood of mammal detection in the area. In each of these identified places, a single camera trap unit (Reconyx hyperfire; Sony p32; Scoutguard 565F) was placed. Camera traps were installed 45 cm above the ground on tree trunks, at least 2 m away from the trail and were often aimed perpendicular to the trail. Each unit was programmed to take images every 60 s when triggered, and were active at all locations for 24 h each day. The minimum and maximum distances between two camera traps were 330 and 2100 m, respectively, but correspond to a greater travel distance on the ground due to terrain.

#### 2.2.2 Data Management

The camera trap images were sorted by two people to improve confidence in classifications. Images were categorized by species, as humans, livestock, blanks, and unidentified. Images of the same species capture in one camera traps were considered to be independent if they were taken at least 1 hour apart (Thorn et al. 2022). To define independent images from two different camera traps, we analyzed the time stamp on the images, the distance between camera traps, and the direction of movement of the species. For example, two photographs of the same species captured in closedly located camera traps were considered single animal if they were captured around the same time and were traveling in the same direction. The independent images were then tagged up to the species level in the application digiKam (digiKam, 2021). Small species that were difficult to identify up to the species level were combined into groups. For example, Nepalese field mouse *Apodemus gorkha* and fawn‐colored mouse *Mus cervicolor* were clumped together as *Mouse* spp. The tagged photos were then imported in (R Core Team 2021), and single species detection histories were created with the function ‘*dethist*’ using R package ‘*camtrapR*’ (Niedbella et al. 2016). These individual detection histories were combined to create an aggregated detection history that included detection and non‐detection data for all species across all sites and served as the input file for our multi‐species occupancy analysis.

#### 2.2.3 Covariates

Given that elevation has been known to influence species distribution and occupancy (McCain and Grytnes 2010), we hypothesized that elevation and ruggedness of the landscape would have significant effect on species occupancy and species richness (Bhattarai and Vetaas 2003; Owen 1990). Variation in canopy cover at our camera trap locations made it an important covariate as it is known to influence species richness positively. Thus, we hypothesized there would be a positive influence of tree canopy cover on the occupancy and richness of mammals in the area. Human disturbance is known to have a negative impact on the species richness and occupancy of mammals (Barber‐Meyer et al. 2013; Murphy and Romanuk 2014), except for a few species like leopard and jungle cat (Athreya et al. 2013; Wu et al. 2020). While the area has little human disturbance due to the absence of permanent roads or other man‐made facilities, there are several local trails which connect Sikles village in the west of the study area to the broader Annapurna trekking circuit at the eastern edge of the study area. We hypothesized that human disturbance has a negative impact on occupancy and species richness due to the presence of livestock and humans. Table 1 contains information on the covariates and our corresponding hypothesis. All covariates were standardized to the mean of 1 and standard deviation of 0 prior to analysis.

**TABLE 1:** Covariates considered for analysis based on their potential influence on the probability of occupancy, detection and species richness. Assumptions provide the hypotheses concerning corresponding covariates.

Covariate	How is it defined?	Source	Assumptions
Elevation	Mean elevation within 100 m radius around the camera trap station	Shuttle Radar Topography Mission (SRTM) files Farr et al. (2007)	We assumed that the mammal occupancy and richness would be influenced by elevation quadratically (Feng et al. 2021)
Terrain ruggedness	Mean ruggedness within 100 m radius around the camera trap station	Used ArcGIS 10.7 to convert the SRTM raster files to Terrain Ruggedness Index (TRI) rasters (Riley, DeGloria, and Elliot 1999)	Terrain ruggedness positively influences occupancy and species richness of mammals (Einoder et al. 2018; Lamichhane et al. 2021)
Canopy cover	Mean canopy cover within 100 m radius around the camera trap station	Global 2010 Tree Cover (30 m) (Hansen et al. 2013)	Occupancy and species richness is positively influenced by canopy cover (Andrade‐Núñez and Mitchell Aide 2010; Penjor et al. 2021)
Human disturbance	Number of independent human and livestock images from the individual camera trap locations	Used independent camera trap images of human and livestock	Human disturbance negatively influences occupancy and species richness of mammals (Murphy and Romanuk 2014; Penjor et al. 2021)

#### 2.2.4 Statistical Modeling

We considered two processes, the ecological process and observation process, which were governed by Bernoulli and Binomial distributions, respectively. The ecological process is represented by:

(1) $$z_{\mathrm{ik}}\sim \text{Bernoulli}\left(\psi_{\mathrm{ik}}*w_{k}\right)$$

where $z_{\mathrm{ik}}$ indicates if site i is occupied by species k ($z_{\mathrm{ik}}=1$ when occupied, 0 otherwise). The occupancy probability $\psi_{\mathrm{ik}}$ denotes probability species k will occupy site i (dependent on‐site conditions), and $w_{k}$ accounts for undetected species. The observation process is represented by:

(2) $$y_{\mathrm{ik}}\sim \text{Binomial}\left(p_{k}*z_{\mathrm{ik}},n_{i}\right)$$

Where $y_{\mathrm{ik}}$ is the number of times species k was observed at site i during $n_{i}$ attempts to observe the species at that site. The probability to detect species k on each attempt is given by $p_{k}$. It was assumed that the probability of detection for individual species was constant across sites and occasions (Kéry and Royle 2015).

We included covariates in our model to estimate species occupancy at each site using the logit link function:

$$\text{Logit}\left(\psi_{\mathrm{ik}}\right)=\beta_{0k}+\beta_{1k}*X_{1i}+\beta_{2k}*X_{2i}+\ldots +\beta_{\mathrm{mk}}*X_{\mathrm{mi}}$$

Where $\beta_{0k}$ is the intercept of the equation, defined by $\beta_{0k}\sim \text{Normal}\left(\mu_{0k},\sigma_{0k}^{2}\right)$,

$\beta_{\mathrm{mk}}$ is the slope associated with the site covariate m, defined by $\beta_{\mathrm{mk}}\sim \text{Normal}\left(\mu_{\mathrm{mk}},\sigma_{\mathrm{mk}}^{2}\right)$, and.

$X_{\mathrm{mi}}$ is the value of covariate m at site i.

As we are also modeling the possible presence of any undetected species, we included $w_{k}$ in the equation (1) which is governed by the Bernoulli distribution:

$$w_{k}\sim \text{Bernoulli}\left(\Omega \right)$$

where Ω is the augmentation parameter. Indicator $w_{k}$ will take the value 1 if species k is detectable, and 0 if not. Augmentation was applied with a maximum of *n* = 21 possible undetected species, equal to the total number of species already detected. We considered this to be small enough to be biologically plausible to our study area but large enough also to not constrain the resulting species richness estimate for the area (Yamaura et al. 2011). Species richness was obtained by

$$N=\sum_{i=1}^{n}w_{i}$$

These derived richness estimates were then used to understand how species richness is influenced by covariates by fitting linear models. Coefficients of slope were examined to interpret the influence of these covariates on mammalian species richness at each individual sites.

We fit the above Bayesian hierarchical model using JAGS (Plummer 2003) via the R package *‘jagsUI’* (Kellner 2021). Our priors were defined as weakly informative and obtained from a uniform, beta, or normal distribution with uniform hyperparameters (Hobbs and Hooten 2015). We used three Markov chain Monte Carlo (MCMC) chains of 100,000 iterations each, thinned by 60. The first 5000 iterations were discarded as burn‐ins. The posterior plots were created using the R package ‘*mcmcOutput*’ (Meredith 2021). We assumed model convergence based on diagnostic traceplots and R‐hat values of 1.1 (Gelman and Rubin 1992).

## 3 Results

We used data from 45 cameras for a total of 4345 trap nights and 15,326 separate photos, with images consisting of wildlife (16.58%, *n* = 2541), human/livestock (3.24%, *n* = 497), unidentified (0.52%, *n* = 80), and blank/false triggers (79.66%, *n* = 12,208). The wildlife photographs comprised 1481 mammals (34.08%), the majority of which were barking deer (42.50%, *n* = 623) or leopard cats *Prionailurus bengalensis* (16.10%, *n* = 238). The two main carnivores in the area, the common leopard and the clouded leopard, were detected on 84 (5.70%) and 7 (0.50%) occasions, respectively. Northern treeshrew *Tupaia belangeri* was only detected once (0.07%) during the entire survey period, which was also the species' westernmost record worldwide. Two species were detected only once, while 9 species were detected twice or more (Table 2).

**TABLE 2:** Category of mammals based on their detection frequency. Measuring species richness while accounting for imperfect detection allows for an estimate of the number of animals that went undetected during the survey, described further in the section ‘Species Richness’.

Detection category	Number of detections	Species count
Abundant	> 100	3
Common	11–100	8
Uncommon	2–10	8
Rare	1	2
Undetected	0	5

### 3.1 Community and Species‐Specific Occupancy and Detection Probabilities

The probability of occupancy differed greatly between species. The maximum probability of occupancy was 0.8 for barking deer, and the lowest was 0.1 for several species, including northern treeshrew and yellow‐bellied weasel *Mustela kathiah* . The occupancy estimates for the area's two largest predators, the common leopard and clouded leopard, were 0.709 (95% CRI: 0.535–0.871) and 0.091 (95% CRI: 0.019–0.236), respectively. The detection probability was lowest for northern treeshrew and yellow‐bellied weasel (0.004, 95% CRI: 0.0003–0.015) whereas highest for northern muntjac (0.189, 95% CRI: 0.176–0.202). Table 3 shows the species‐specific detection and occupancy probability. For the purportedly present species that went unnoticed, the probability of occupancy and detection were 0.241 (95% CRI: 1.93e‐04–0.973) and 0.0329 (95% CRI: 1.91e‐04–0.193), respectively.

**TABLE 3:** Estimates of the probability of occupancy (ψ) for all confirmed species.

Species	Scientific name	Naïve detection probability	Probability of detection p (95% CRI)	Naïve occupancy probability	Probability of occupancy ψ (95% CRI)
Clouded leopard	*Neofelis nebulosa*	0.002	0.02 (0.01–0.04)	0.07	0.09 (0.02–0.24)
Crab‐eating mongoose	*Herpestes urva*	0.003	0.03 (0.02–0.05)	0.11	0.10 (0.03–0.21)
Squirrel	*Dremomyx/Callosciurus* spp.	0.002	0.02 (0.01–0.04)	0.09	0.13 (0.04–0.29)
Pika	*Ochotona* spp.	0.001	0.01 (0.001–0.03)	0.02	0.15 (0.01–0.81)
Yellow‐bellied weasel	*Mustela kathiah*	0.0002	0.004 (0.0003–0.02)	0.02	0.12 (0.01–0.87)
Northern treeshrew	*Tapia belangeri*	0.0002	0.004 (0.0003–0.02)	0.02	0.20 (0.01–0.88)
Asiatic wild dog	*Cuon alpinus*	0.001	0.004 (0.001–0.014)	0.04	0.22 (0.02–0.83)
Spotted linsang	*Prionodon pardicolor*	0.001	0.004 (0.0004–0.02)	0.04	0.25 (0.03–0.90)
Masked palm civet	*Paguma larvata*	0.005	0.02 (0.01–0.03)	0.22	0.25 (0.11–0.46)
Mouse	*Mus/Apodemus spp*.	0.02	0.06 (0.05–0.07)	0.36	0.33 (0.20–0.48)
Assam macaque	*Macaca assamensis*	0.01	0.03 (0.02–0.05)	0.36	0.36 (0.21–0.53)
Malayan porcupine	*Hystrix brachyura*	0.01	0.02 (0.01–0.03)	0.33	0.39 (0.23–0.61)
Common goral	*Naemorhedus goral*	0.002	0.005 (0.002–0.01)	0.13	0.42 (0.11–0.93)
Nepal gray langur	*Semnopithecus schistaceus*	0.001	0.004 (0.001–0.01)	0.11	0.43 (0.09–0.94)
Large Indian civet	*Viverra zibetha*	0.02	0.05 (0.04–0.06)	0.49	0.51 (0.35–0.67)
Asiatic black bear	*Ursus thibetanus*	0.002	0.006 (0.002–0.01)	0.20	0.52 (0.19–0.94)
Yellow‐throated marten	*Martes flavigula*	0.03	0.05 (0.04–0.05)	0.67	0.69 (0.54–0.84)
Common leopard	*Panthera pardus*	0.03	0.03 (0.03–0.04)	0.62	0.71 (0.53–0.87)
Leopard cat	*Prionailurus bengalensis*	0.06	0.09 (0.08–0.1)	0.67	0.71 (0.56–0.85)
Himalayan serow	*Capricornis thar*	0.01	0.02 (0.01–0.02)	0.56	0.73 (0.51–0.93)
Barking deer	*Muntiacus vaginalis*	0.15	0.19 (0.18–0.20)	0.80	0.82 (0.69–0.92)

*Note:* The common name, scientific name, naïve detection probability, estimated detection probability, naïve occupancy probability and estimated occupancy probability. Barking deer was the most frequently detected species while the yellow‐bellied weasel was the least frequently detected mammal species in the area.

### 3.2 Species Richness

The mean species richness estimated by the model was 25.60 species (95% CRI = 21–36). The posterior mean estimate for the most important model parameters is provided in Table 4.

**TABLE 4:** Estimates of the parameters from the model: mean community occupancy (ψ), mean community detection probability (p), and derive parameter species richness (*N*). *R* is the Gelman‐Rubin ratio.

Parameter	Mean	SE	2.5%	Median	97.5%	*R*
N	25.60	3.96	21	25	36	1.001
Ψ	0.28	0.09	0.08	0.28	0.46	1.008
*p*	0.02	0.01	0.01	0.02	0.03	1.002

**FIGURE 2:**
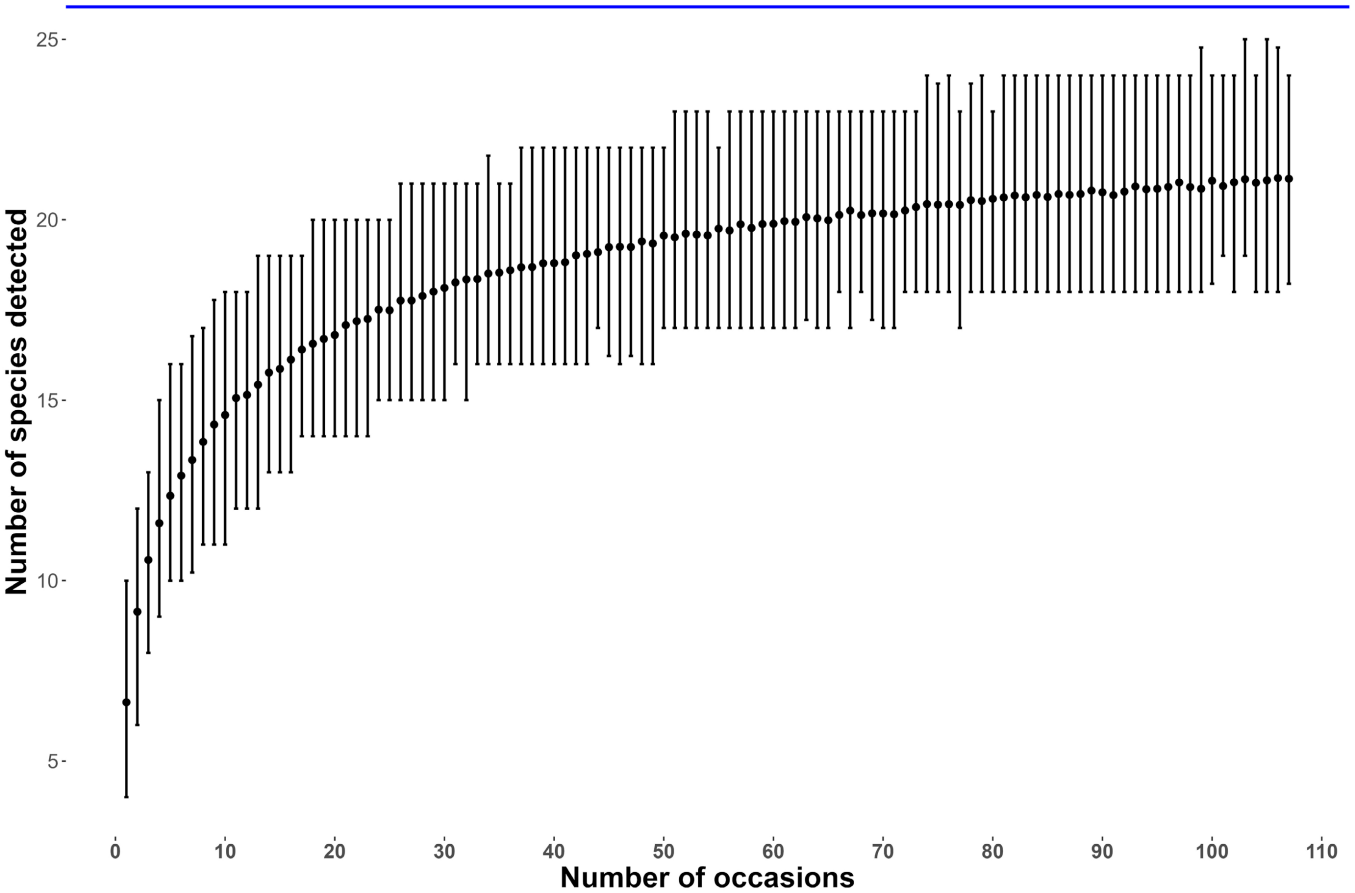
Species accumulation curve with estimated species richness indicated by the blue horizontal line. The vertical line indicate standard deviation from the calculated mean species richness from all sites.

A species accumulation curve provides information on the process of species detection on each survey occasion (Figure 2). The curve indicates that the asymptote was not reached at the end of the survey.

### 3.3 Covariates' Influence on Occupancy and Species Richness

**FIGURE 3:**
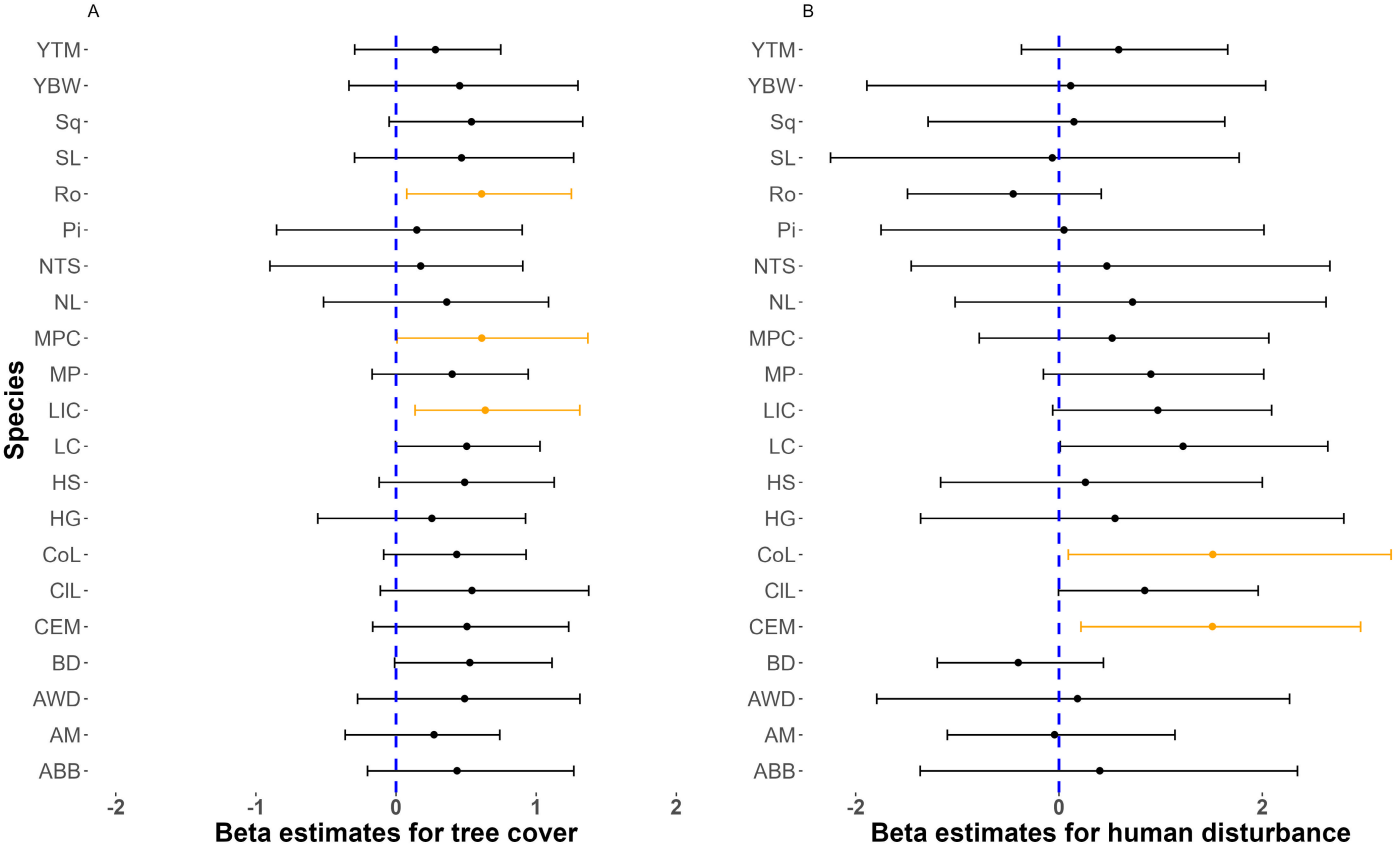
Individual species' response to tree cover and human disturbance in ACA. The blue dashed lines indicate zero, which represents no influence of covariates. The black points are the posterior means of the individual species' response, and the black lines are 95% credible intervals. The orange point and horizontal lines represent species with a strong response whose 95% credible interval (orange horizontal line) do not overlap zero. Species acronyms include ABB (Asiatic black bear), AM (Assamese macaque), AWD (Asiatic wild dog), BD (barking deer), CEM (crab‐eating mongoose), ClL (clouded leopard), CoL (common leopard), HG (Himalayan goral), HS (Himalayan serow), LC (leopard cat), LIC (large Indian civet), MP (Malayan porcupine), MPC (masked palm civet), NL (Nepal langur), NTS (northern treeshrew), Pi (pika), Ro (rodent), Sq (squirrel), YBW (yellow‐bellied weasel), and YTM (yellow‐throated marten).

Canopy cover positively influenced occupancy of all species, with the strongest evidence for large Indian civet (β = 0.568, 95% CRI: 0.062–1.199), masked palm civet (β = 0.592, 95% CRI: 0.002–1.347), and rodents (β = 0.596, 95% CRI: 0.078–1.272). Human disturbance influenced the probability of occupancy of common leopard (β = 1.697, 95% CRI: 0.135–3.499) and crab‐eating mongoose occupancy (β = 1.656, 95% CRI: 0.285–3.190) positively. The influence of tree cover and human disturbance on the occupancy of all animal species in our research region is depicted in Figure 3.

The canopy cover was greater than 65% at four of the six sites that recorded at least 10 species of mammals. Further analysis revealed a statistically significant positive relationship between tree canopy cover and mammalian species richness. Species detections increased between the elevations of 2000 and 2500 m, with six of the seven camera traps in that elevation range detecting at least 10 mammal species. Modeled species richness showed strong quadratic influence of elevation on mammalian species, which was estimated to be increasing to a maximum around approximately 2100 and declining thereafter. Human disturbance had surprisingly positive relationship with mammal species richness in the area (Table 5).

**TABLE 5:** Regression coefficients of modeled species richness against each of the considered covariate.

Covariates	*β*	*se*	95% CI		t	*p*
			Lower	Upper		
Elevation	−1.210	0.423	−2.039	−0.381	−2.858	0.007
Elevation^2^	−1.750	0.381	−2.497	−1.003	−4.588	4.00e‐5
Ruggedness	0.258	0.550	−0.820	1.336	0.470	0.641
Tree cover	1.908	0.469	0.989	2.827	4.071	1.95e‐4
Disturbance	1.339	0.513	0.334	2.344	2.613	0.012

## 4 Discussion

Species richness is an essential parameter indicative of a region's biotic community. Despite this, studies aiming to estimate this important parameter do not incorporate imperfect detection (Guillera‐Arroita, Kery, and Lahoz‐Monfort 2019; MacKenzie et al. 2017). MSOMs incorporate imperfect detection within their statistical framework, making them an ideal tool to estimate species richness (Guillera‐Arroita, Kery, and Lahoz‐Monfort 2019; Kéry and Royle 2015). However only rarely have they been used to evaluate species richness in the Himalayan region, which is an important repository of mammalian assemblage (but see Regmi et al. 2023; Thapa et al. 2022). Furthermore, despite the well‐known influence of environmental factors on mammalian species, there has been virtually no effort made to discern these influences (but see Regmi et al. 2023). Given the rapid pace of environmental change across the world, it is crucial to create baselines to efficiently monitor ecological communities in the future. Here, we used single‐season MSOMs to estimate species‐specific detection and occupancy probabilities, as well as mammalian species richness in ACA, Nepal, and the factors that influence these parameters.

The probability of occupancy (0.1–0.8) varied substantially among member species, as would be expected in an ecological community where each species' rarity is on a continuum (May 1975). Due to few detections, occupancy estimates for species such as the Nepal langur Sand Asiatic black bear varied greatly. The estimates of muntjac and leopard cat show that variability decreases with increasing detection frequency. Even after incorporating imperfect detection, species with high detection frequencies, such as barking deer (naïve occupancy = 0.80, estimated occupancy = 0.82) and leopard cat (naïve occupancy = 0.67, estimated occupancy = 0.71), did not show a large change in the probability of occupancy compared to the naïve estimates. However, the increase was large for some species, such as Himalayan serow (naïve occupancy = 0.55, estimated occupancy = 0.73). While there was a large increase in the probability of occupancy for other species such as dhole and spotted linsang, there was also high uncertainty in the estimate due to extremely few detections (two each for both species).

In general, the detection probability was low for all species. The highest was for barking deer, at 0.19 (95% CRI: 0.176–0.202). Similarly low estimate of detection probability (< 0.2) have been recorded from Dhorpatan Hunting Reserve and Dadeldhura districts (Regmi et al. 2023; Thapa et al. 2022). Surveys in Bangladesh, Bhutan, and India gave estimates of 0.09, 0.12, and 0.22, respectively (Chaudhary et al. 2022; Penjor et al. 2021; Rahman et al. 2021). All these regions have similar habitat, geography, and co‐inhabiting species, making this comparison relevant. As a result, we believe that the low detection probability is the norm rather than exception. In the present study, preferential placement of cameras targeting felid habitat will influence the detection rate of clouded leopard and leopards, as well as likely influencing the detection probability of prey, such as barking deer, or other mammals in the community (Boron et al. 2019; Kolowski and Forrester 2017; Regolin et al. 2017). For potentially missed species, the probability of occupancy and detection were 0.254 (95% CRI: 1.93e‐15–0.919) and 0.001 (95% CRI: 3.4e‐08–0.016), respectively. The probability of occupancy is relatively high but there is also a high uncertainty in this parameter while the probability of detection is unsurprisingly very low and highly uncertain. These low values of the probability of occupancy and detection of the supposedly missed species possibly explains why these species were never detected.

According to our findings, the estimated species richness for the ACA, after accounting for imperfect detection, is 26 species (mean = 25.60, 95% CRI: 21–36). The species accumulation curve is gradually leveling off, but the asymptote was not reached within the length of the survey (Figure 3). The richness estimate implies that our survey missed 19% of the total species. Our understanding of the distribution of mammals in Nepal based on established literature indicates that 6–7 species are likely to be missing (Baral and Shah 2008). As a result, our species richness estimation represents an acceptable depiction of the area. In fact, our study was followed up by two surveys in 2018 and 2021, each of which yielded one previously unrecorded species i.e., musk deer *Moschus* spp. and wild boar *Sus scrofa* . Because musk deer prefer areas of higher altitudes with rhododendron woodland and scrubs (Timmins and Duckworth 2015), our 2017 survey effort possibly missed the species due to lower survey effort at high elevations. The wild boar was supposedly locally extinct for approximately five decades but is now attempting to repopulate the area. During our 2017 study, we did observe wild boar evidence such as rootlings, but their occupancy was likely too low or the species was too timid to come on cameras, or both. Other species that may have escaped observation include the beech marten *Martes foina* , jungle cat *Felis chaus* , red fox *Vulpes vulpes* , large‐toothed ferret badger *Melogale personata* , and Siberian weasel *Mustela sibirica* (Baral and Shah 2008).

Being a conservation area, visitation by humans is integral to the area and mammalian community inhabiting the area essentially gets influenced. The area is visited by local tourists and NTFP collectors during the winter and early spring (January–March). Human activities, contrary to our assumptions, positively influenced the occupancy of two species, the common leopard, and the crab‐eating mongoose. While common leopards are generally known to be elusive and avoid human disturbance (Ngoprasert, Lynam, and Gale 2007; Paudel and Kindlmann 2012), they can adapt to varying levels of human activity, even excelling in areas with moderate to high human disturbance in some cases (Athreya et al. 2013; Gubbi, Sharma, and Kumara 2020). A few livestock corrals also operate inside the study area that might provide dietary supplement for leopards potentially explaining its positive relationship with human disturbance. Although discussion with herders did not indicate recent cases of livestock depredation by the species in recent years. The crab‐eating mongoose is not a well‐studied animal, but it is thought to react negatively to human disturbance (Sharma et al. 2021). We believe that the presence of livestock possibly influence the habitat diversity positively at the local level (Porras et al. 2016), helping enhance the occupancy of some species which has been observed in Dhorpatan Hunting Reserve (Regmi et al. 2023). While mesopredators like leopard cat, and possibly large Indian civet, have been shown to benefit from moderate to high levels of human disturbance (Villalva, Palomares, and Zanin 2024; Wu et al. 2020) we did not find any such evidence in ACA. The impact of human disturbance on modeled species richness was significantly positive, in contrast to suggestive evidence of a smaller effect in models fit using only observed species richness $\left(\beta =0.610,p:0.213\right)$. Incorporation of imperfect detection while modeling species richness possibly made the difference here. However, despite the apparent positive influence of human disturbance on mammalian species richness, a closer look revealed that humans and wild mammals were active during different time periods and the temporal overlap was not significant $\left(D_{\mathrm{hat}}=0.56\right)$. While humans predominantly used the time around the noon while these species, especially the predators, primarily used the time between sunset and sunrise. Surveys during periods of high human activities might provide a better picture of how human disturbance influences species richness in the area and if there is a threshold of human activity below which it might benefit wildlife. However, when comparing our results to those from highly disturbed environments, caution should be exercised.

Elevation influenced the modeled species richness negatively meaning generally decreasing trend of species richness with increasing elevation. The coefficient for the quadratic elevation term is also negative, suggesting the steeper decline after a certainn elevation, which is ~2160 m in our area. While the unimodal relationship between the species richness and elevation is generally true, researches show this relationship to be much more complicated showing various patterns like decreasing with increasing elevation, low‐elevation plateau, low‐elevation plateau with mid‐peak, and mid‐peak (McCain and Grytnes 2010). Also, these trends often vary by species/taxonomic groups and might not be applicable for all species. Species richness of an area is also dependent on the time of the survey. Thus, a continuous, multi‐year survey would throw further light on whether the relationship between elevation and species richness remains the same or change between seasons.

Finally, tree canopy cover had a strong beneficial influence on species richness, which is not surprising considering that animals generally prefer forests with a good canopy (Andrade‐Núñez and Mitchell Aide 2010; Yue et al. 2015). Species‐specific response to tree cover was generally insignificant, except for large Indian and masked palm civets, whose probability of occupancy was influenced positively by tree canopy cover. We anticipated species like clouded leopard and Himalayan serow to have positive association with areas having greater tree canopy cover (Bhattacharya et al. 2012; Penjor et al. 2021; Tan et al. 2017), however this was not observed, which is difficult to understand and will most likely require more research. Additionally we expected species such as goral and Himalayan serow to have positive association with TRI due to their preference to more rugged, steeper areas (Duckworth, MacKinnon, and Tsytsulina 2008; Duckworth and MacKinnon 2008), there was no evidence to support these predictions.

Our research establishes a baseline for species richness and occupancy of mammals in a typical subtropical‐temperate forested environment in Nepal's mountainous protected area and reaffirms the significance of incorporating imperfect detection within the study design and analysis. Because our study area is well protected, relative to other mountainous areas in Nepal, direct comparisons must be made with caution, particularly when it comes to the issue of human disturbance. We hope that our findings can be used to assess the effectiveness of conservation monitoring programs in the coming years, as species richness surveys provide an important baseline for conservation planning. Further surveys in different seasons (summer, rainy) would help establish if and how the estimates alter with season and to corroborate the influence of environmental factors. We recommend the use of MSOMs that incorporate imperfect detection into survey design and analysis in multi‐taxa research. Multi‐year surveys are highly recommended to determine how the status of the species, as well as the makeup and richness of the community, vary over time. Such efforts will greatly supplement existing research efforts and contribute significantly to future conservation projects in the region.

## Author Contributions

**Yadav Ghimirey:** conceptualization (lead), data curation (lead), formal analysis (lead), funding acquisition (lead), investigation (equal), methodology (lead), project administration (equal), validation (equal), visualization (equal), writing – original draft (lead), writing – review and editing (lead). **Raju Acharya:** conceptualization (equal), funding acquisition (equal), investigation (equal), project administration (equal), writing – review and editing (equal). **Jeffrey Mintz:** formal analysis (equal), validation (equal), writing – review and editing (equal).

## Conflicts of Interest

The authors declare no conflicts of interest.

## Data Availability

Data are provided as Supporting Information.
